# Antihypertensive utilization patterns among pregnant persons with pre-existing hypertension in the US: A population-based study

**DOI:** 10.1371/journal.pone.0306547

**Published:** 2024-07-03

**Authors:** Yanning Wang, Nicole E. Smolinski, Celeste Ewig, Thuy Nhu Thai, Tony S. Wen, Almut G. Winterstein

**Affiliations:** 1 Department of Pharmaceutical Outcomes and Policy, College of Pharmacy, University of Florida, Gainesville, FL, United States of America; 2 Department of Health Outcomes and Biomedical Informatics, College of Medicine, University of Florida, Gainesville, FL, United States of America; 3 Center for Drug Evaluation and Safety, University of Florida, Gainesville, FL, United States of America; 4 Department of Population Medicine, Harvard Medical School and Harvard Pilgrim Health Care Institute, Boston, MA, United States of America; 5 Department of Obstetrics and Gynecology, College of Medicine, University of Florida, Gainesville, FL, United States of America; 6 Department of Epidemiology, College of Medicine and College of Public Health and Health Professions, University of Florida, Gainesville, FL, United States of America; HT Ong Heart Clinic, MALAYSIA

## Abstract

**Background:**

Hypertension among persons with childbearing potential is on the rise. Maintaining proper blood pressure during pregnancy is vital to prevent maternal and neonatal complications. Yet, limited evidence on the risk-benefit of various antihypertensives presents challenges for informed decision-making during this critical period. This study aimed to examine the utilization patterns of different classes of antihypertensives among persons with pre-existing hypertension before, during, and after pregnancy.

**Methods:**

We used MarketScan® Commercial Database 2011−2020 to analyze antihypertensive utilization among pregnant persons aged 12 to 55 identified via a validated algorithm. Pre-existing hypertension was defined as ≥1 inpatient or ≥2 outpatient encounters for hypertension within the 180 days preceding the LMP. Antihypertensive utilization was described during target periods: 0–3 months (0-3M) before pregnancy, 1st/2nd/3rd trimester (T1/2/3), 0-3M, and 4-6M after pregnancy.

**Results:**

We identified 1,950,292 pregnancies, of which 20,576 (12,978 live and 7,598 non-live) had pre-existing hypertension. Both groups had similar antihypertensive use (80.1% and 81.0%, respectively) during the 6 months before pregnancy (baseline). For live-birth pregnancies, 13.9% of baseline users discontinued treatment during pregnancy, while 28.9% of non-users initiated antihypertensives during pregnancy, and 17.2% started postpartum. Before pregnancy, the predominant antihypertensives included thiazide diuretics (21.9%), combined α- and β-blockers (18.4%), and dihydropyridines (16.2%). During pregnancy, thiazide diuretics, cardioselective β-blockers, and ACE inhibitors declined (T3: 3.0%, 4.2%, and 0.8%). Dihydropyridine use was steady during pregnancy, but preference shifted from amlodipine to nifedipine in T3 (2.2.% vs.10.8%). Central α2‐agonists increased during pregnancy (up to 15.2% in T3) compared to both pre- (9.8%) and post-pregnancy (5.7%). ARBs mirrored ACE inhibitors, with less than 1% utilization in later trimesters. Combination agents dropped from 10.8% pre-pregnancy to 0.8% in T3, then rebounded to 7.3% post-pregnancy.

**Conclusion:**

Research is warranted to evaluate the choice of antihypertensives and optimal timing to switch to safer alternatives, considering maternal and fetal outcomes.

## Introduction

Hypertensive disorders in pregnancy (HDP) are common complications that affect about one in seven hospital deliveries and contribute to 7% of pregnancy-related deaths in the United States (US) [1, 2]. Within the spectrum of HDPs, chronic hypertension in pregnancy is a specific concern. This condition is defined as hypertension that predates pregnancy (pre-existing hypertension) or is first diagnosed before 20 weeks of gestation.

Chronic hypertension in pregnancy is associated with increased risks of adverse maternal and fetal outcomes [3, 4]. According to national vital statistics, its prevalence has been growing in the US, impacting 2.7% of live births in 2022 [5]. Historically, the treatment approach for chronic hypertension during pregnancy in the US was notably conservative. The antihypertensive treatment threshold was set at a blood pressure (BP) of 160/110 mmHg, higher than the 140/90 mmHg recommended for the general non-pregnant population, largely due to limited evidence on the risk-benefit of antihypertensive treatment during pregnancy [3, 4, 6, 7]. However, the recent findings from the Chronic Hypertension and Pregnancy (CHAP) study have prompted a shift [8]. The American College of Obstetricians and Gynecologists (ACOG) revised the threshold to 140/90 mmHg, recommending the continuation of antihypertensive treatments from pre-pregnancy unless specific safety concerns emerge [9]. While monotherapy of labetalol, methyldopa, and nifedipine are the recommended first-line antihypertensive agents during pregnancy, other medications might be considered based on individual patient conditions [3, 4, 6, 7, 10]. However, there’s a consensus against using renin-angiotensin system-acting agents, such as ACE inhibitors, ARBs, and direct renin inhibitors, especially during the late stages of pregnancy, due to adverse effects on fetal kidney development and function [3, 4, 7, 11–15]. Of note, the evidence supporting these recommendations remains scanty, complicating the management of chronic hypertension in patients of childbearing age. Importantly, prior US-based studies have not delved deep into antihypertensive use during pregnancy among patients with pre-existing hypertension [16–18].

Understanding utilization patterns can pave the way for improved management strategies for chronic hypertension during pregnancy and define key areas for further inquiry. Our study sought to address this by investigating antihypertensive utilization patterns in this patient population, spanning the periods before, during, and after pregnancy. Through this, we aimed to offer real-world evidence that could guide future research on the best antihypertensive choices or when to transition to safer alternatives.

## Materials and methods

### Data source and study cohort

We used the Merative™ MarketScan® Commercial Database to analyze antihypertensive utilization among pregnancy episodes that ended between 2011 to 2020. This database comprises a national sample of individuals with employer-sponsored health insurance and provides details on patient demographics, insurance enrollment, diagnoses, procedures received during inpatient and outpatient services, and dispensed outpatient prescription medications. We adapted a previously validated algorithm to identify pregnancy episodes in individuals aged 12 to 55 years, resulting in both live and non-live births (ectopic, spontaneous or induced abortions, and stillbirths) [19]. We required continuous health plan enrollment from 180 days before the last menstrual period (LMP) until 180 days after the pregnancy end date. Pre-existing hypertension was defined as having ≥1 inpatient or ≥2 outpatient encounters for essential hypertension (**S1 Appendix**) 180 days before LMP. The University of Florida Institutional Review Board considered the project exempt from human subjects research review due to the use of de-identified data.

### Antihypertensive exposures

Our study used the National Drug Code (NDC) on outpatient pharmacy claims to define outpatient antihypertensive exposures. We selected the antihypertensive agents approved for this indication in the US using RED BOOK®. Medications included in the study are listed in **S2 Appendix**. Antihypertensive exposure was defined as having ≥1 outpatient pharmacy claim during a targeted pregnancy-related period. We determined the time of exposure based on the medication dispensation date and considered the duration of exposure periods based on prescription fill date and dispensed days’ supply in a sensitivity analysis.

### Patient characteristics

We categorized patients based on their demographics and selected clinical characteristics, focusing on those who used antihypertensives in the 180-day pre-pregnancy period (baseline). Age groups were defined according to the National Center for Health Statistics age reporting categories. Diagnosis and procedure codes from medical encounters were used to compile patients’ clinical history, as detailed in **S1 Appendix**.

### Antihypertensive utilization pattern

To provide a detailed understanding of antihypertensive utilization, we summarized utilization patterns in five pregnancy-related periods: the 3-month pre-pregnancy period (90 days before LMP to the day prior to the LMP), first trimester (T1: LMP through day 90 of pregnancy), second trimester (T2: day 91 to day 180 of pregnancy), third trimester (T3: day 181 to the day before delivery), 3-month postpartum (delivery to day 89 post-delivery), and 4-6-month postpartum (day 90 to day 179 after delivery).

We calculated exposure prevalence as the number of pregnancies with exposure during the relevant assessment window divided by the total number of assessed pregnancies. Due to the short gestation duration of pregnancies with non-live outcomes, our analysis focused on live-birth pregnancies to describe the utilization pattern. We summarized the exposure by therapeutical class and active ingredient (**S2 Appendix**). If a pregnancy was exposed to more than one category of interest, each category was considered individually. In addition, we reported the prevalence of using combination medications and exposure to polytherapy―receiving antihypertensives from different classes in a given pregnancy-related period.

To understand medication initiation and transition patterns comprehensively, we classified medications into five broad therapeutic classes: Renin-angiotensin-system (RAS)-acting agents, beta-blockers, calcium channel blockers (CCB), diuretics, and others. Emphasis was placed on three preferred drugs: labetalol, nifedipine, and methyldopa, which were treated as standalone categories to quantify medication initiation and switching.

Data management and analyses were conducted using SAS® 9.4 (Cary, NC).

## Results

We identified 1,950,292 (1,427,819 live and 522,473 non-live) pregnancies ending between 2011 and 2020 with the required health insurance coverage. Of these, there were 20,576 (12,978 live-birth and 7,598 non-live) pregnancies with pre-existing hypertension, representing 0.9% of live-birth and 1.5% of non-live pregnancies. During the ten-year study period, the prevalence of pre-existing hypertension increased for both live-birth (from 0.8% to 1.1%) and non-live (from 1.2% to 1.6%) pregnancies.

Around 80.4% of the pregnancies with pre-existing hypertension used antihypertensives during the 6-month baseline period (**Table 1**). Pregnancies occurred at a higher age than reported in national data, with over half of persons aged 35 and above. The baseline antihypertensive users were older than the non-users, with a median age of 35 years compared to 33 years. Baseline use of antihypertensives increased with maternal age, from 49.3% in persons aged 12–19 years to 86.0% in those aged 44–55 years. There was no difference in live birth and stillbirth rates between baseline users and non-users. Across all pregnancies, one in five had diagnoses of lipid metabolism disorders, 18.3% of the cohort had pregestational diabetes, 15.9% had anxiety disorders, and 10.4% had depression. Only 1% to 2% had chronic kidney disease, ischemic heart disease, or congenital heart failure.

**Table 1:** Characteristics of pregnancies from patients with pre-existing hypertension, MarketScan® commercial claims databases, 2011–2020.

		Baseline Antihypertensive Use	
	Total	No	Yes
	N = 20,576	N = 4,023	N = 16,553
**Maternal age, median years (IQR)**	35 (7)	33 (8)	35 (8)
12–19	67 (0.3%)	34 (0.8%)	33 (0.2%)
20–24	809 (3.9%)	322 (8.0%)	487 (2.9%)
25–29	2,799 (13.6%)	716 (17.8%)	2,083 (12.6%)
30–34	6,443 (31.3%)	1,282 (31.9%)	5,161 (31.2%)
35–39	6,604 (32.1%)	1,120 (27.8%)	5,484 (33.1%)
39–44	3,109 (15.1%)	445 (11.1%)	2,664 (16.1%)
44–55	745 (3.6%)	104 (2.6%)	641 (3.9%)
**Pregnancy Outcome**			
Live birth	12,978 (63.1%)	2,581 (64.2%)	10,397 (62.8%)
Preterm (Live Birth Only)*	2,847 (21.9%)	518 (20.1%)	2,329 (22.4%)
Spontaneous abortion	5,694 (27.7%)	1,029 (25.6%)	4,665 (28.2%)
Induced abortion	1,221 (5.9%)	287 (7.1%)	934 (5.6%)
Ectopic pregnancy	463 (2.3%)	90 (2.2%)	373 (2.3%)
Stillbirth	220 (1.1%)	36 (0.9%)	184 (1.1%)
**Comorbidities**			
Preeclampsia*	3,416 (26.3%)	631 (24.4%)	2,785 (26.8%)
Disorders of lipid metabolism	4,183 (20.3%)	878 (21.8%)	3,305 (20.0%)
Diabetes	3,767 (18.3%)	738 (18.3%)	3,029 (18.3%)
Anxiety	3,278 (15.9%)	606 (15.1%)	2,672 (16.1%)
Depression	2,150 (10.4%)	446 (11.1%)	1,704 (10.3%)
Chronic Kidney Disease	322 (1.6%)	37 (0.9%)	285 (1.7%)
Ischemic Heart Disease	283 (1.4%)	64 (1.6%)	219 (1.3%)
Congestion Heart Failure	121 (0.6%)	16 (0.4%)	105 (0.6%)
Number of Inpatient Admissions			
1	1,174 (5.7%)	238 (5.9%)	936 (5.7%)
2+	224 (1.1%)	32 (0.8%)	192 (1.2%)
**Healthcare/Medication Utilization** *			
1st trimester Prenatal Care	10,468 (80.7%)	2,041 (79.1%)	8,427 (81.1%)
Antihypertensive Initiation	N/A	745 (28.9%)	N/A
Antihypertensive Discontinuation	N/A	N/A	1,443 (13.9%)

*percentages reported among live-birth pregnancies only

In this cohort, 63.1% of pregnancies resulted in a live birth. Within this subset, over one in four had pre-eclampsia, 21.9% experienced preterm deliveries before 37 weeks of gestation, and 80.7% initiated prenatal care in the first trimester. Among 10,397 pregnancies using antihypertensives at baseline (baseline users), 13.9% discontinued treatment during pregnancy. On the other hand, 28.9% of 2,581 pre-pregnancy non-users initiated antihypertensives during pregnancy, and an additional 17.2% initiated treatment postpartum.

Overall, 69.9% of live-birth pregnancies had prescription fills for at least one antihypertensive three months before LMP (**Table 2** and **S1 Table**, 76.0% considering days’ supply in the sensitivity analysis; **S2 Table**). The most commonly used medication classes and the top antihypertensive in the class were thiazide diuretics (21.9%; hydrochlorothiazide), combined α- and β-blockers (18.4%; labetalol), and dihydropyridines (16.2%; amlodipine), ACE inhibitors (13.0%; lisinopril), and cardioselective β-blockers (11.2%; metoprolol).

**Table 2:** Prevalence of antihypertensive medication exposure before, during, and after pregnancy by class, live birth only (N = 12,978).

	Pre-pregnancy		1st Trimester		2nd Trimester		3rd Trimester*		0-3m Postpartum		4-6m Postpartum	
**Antihypertensive Class**	N	%	N	%	N	%	N	%	N	%	N	%
Beta blockers—combined alpha- and beta-receptor	2,382	18.4%	3,843	29.6%	3,971	30.6%	4,087	31.7%	4,561	35.1%	2,759	21.3%
CCB—dihydropyridines	2,100	16.2%	1,969	15.2%	1,521	11.7%	1,674	13.0%	3,147	24.2%	2,227	17.2%
Central alpha_2_-agonist and other centrally acting drugs	1,276	9.8%	2,209	17.0%	1,989	15.3%	1,877	14.6%	1,311	10.1%	736	5.7%
Thiazide or thiazide-type diuretics	2,846	21.9%	1,762	13.6%	437	3.4%	287	2.2%	1,732	13.3%	1,835	14.1%
Beta blockers—cardioselective	1,453	11.2%	1,019	7.9%	514	4.0%	461	3.6%	890	6.9%	942	7.3%
ACE inhibitors	1,681	13.0%	860	6.6%	118	0.9%	68	0.5%	991	7.6%	1,154	8.9%
ARBs	902	7.0%	533	4.1%	107	0.8%	71	0.6%	525	4.0%	726	5.6%
Diuretics—loop	121	0.9%	86	0.7%	39	0.3%	35	0.3%	426	3.3%	133	1.0%
Diuretics—potassium sparing	280	2.2%	158	1.2%	40	0.3%	27	0.2%	145	1.1%	138	1.1%
Beta blockers—noncardioselective	214	1.6%	153	1.2%	75	0.6%	61	0.5%	107	0.8%	115	0.9%
CCB—nondihydropyridines	181	1.4%	145	1.1%	72	0.6%	60	0.5%	125	1.0%	132	1.0%
Direct vasodilators	104	0.8%	98	0.8%	74	0.6%	84	0.7%	219	1.7%	100	0.8%
Beta blockers—cardioselective and vasodilatory	169	1.3%	145	1.1%	37	0.3%	26	0.2%	80	0.6%	110	0.8%
Diuretics—aldosterone antagonists	128	1.0%	54	0.4%	15	0.1%	9	0.1%	92	0.7%	118	0.9%
Beta blockers—intrinsic sympathomimetic activity	60	0.5%	70	0.5%	52	0.4%	42	0.3%	38	0.3%	31	0.2%
Alpha-1 blockers	9	0.1%	10	0.1%	5	0.0%	2	0.0%	14	0.1%	23	0.2%
Direct renin inhibitor	4	0.0%	4	0.0%	0	0.0%	0	0.0%	2	0.0%	1	0.0%
Other Antihypertensives	0	0.0%	0	0.0%	0	0.0%	0	0.0%	0	0.0%	0	0.0%
**Any Use**	9,036	69.6%	8,499	65.5%	7,455	57.4%	7,355	57.1%	8,651	66.7%	7,433	57.3%
**Combination Product**	1,400	10.8%	809	6.2%	143	1.1%	97	0.8%	787	6.1%	945	7.3%

*N = 12,881; ACE: angiotensin-converting enzyme; ARB: angiotensin receptor blocker; CCB: calcium channel blocker

Throughout pregnancy, the use of thiazide diuretics, cardioselective β-blockers, and ACE inhibitors experienced a decline (T1: 19.9%, 10.7%, and 10.8%, respectively), with the drop becoming more pronounced in the later trimesters (T3: 3.0%, 4.2%, and 0.8%, respectively). Exposures to dihydropyridines slightly decreased during pregnancy (T1: 15.2%; T2: 11.7%; T3: 13.0%), with amlodipine being the most frequent choice in the first trimester but getting replaced by nifedipine in subsequent trimesters. (e.g., T3: 10.8% for nifedipine vs. 2.2.% for amlodipine). Central α2-agonists, mainly methyldopa, had a higher utilization during pregnancy (prevalence: T1 at 14.7%, T2 at 15.4%, and T3 at 15.2%) compared to both pre-pregnancy (9.8%) and post-pregnancy periods (5.7% in the 4–6 month postpartum). ARBs showed a similar pattern as ACE inhibitors–decreasing to less than 1% in the second and third trimesters, then rebounding post-pregnancy. Combination drug prescriptions also fluctuated, dropping from 10.8% before LMP to 0.8% in the third trimester to 7.3% in the 4–6 months postpartum period.

**Fig 1:**
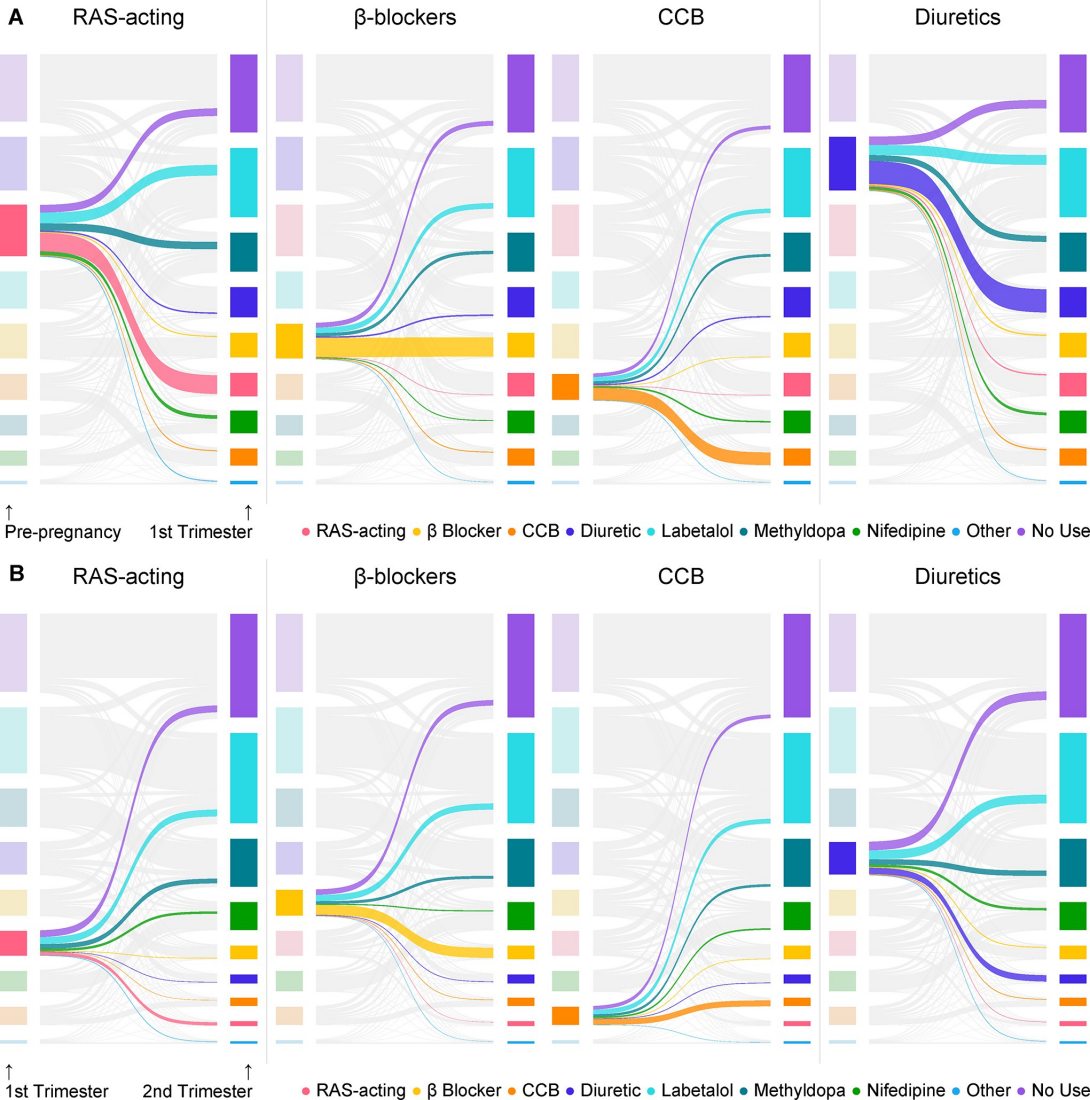
Transition of antihypertensive exposure from pre-pregnancy to the first trimester (A) and from the first trimester to the second trimester (B) among patients with exposures to certain classes of antihypertensives, live birth only.

For those on non-preferred medications before LMP, transitions to other medication or discontinuing therapy were more likely to occur in the first and second trimesters (**Fig 1, S3 and S4 Tables**). Similar switching patterns were observed from pre-pregnancy to the first trimester across drug groups; roughly half of the patients maintained their current drug class, between 30% to 50% transitioned to a preferred agent, and fewer than 20% discontinued antihypertensives. Users of RAS-acting agents and diuretics showed a higher propensity to stop therapy or switch to preferred agents than beta-blocker and CCB users. Of those on RAS-acting agents and diuretics in the first trimester, only 12.9% and 19.2% continued in the second trimester, compared to 40.0% and 32.1% continuation observed for beta-blocker and CCB users. Generally, 75% to 85% of non-users in the current period remained untreated in the subsequent period, except for the third trimester (59%). In the third trimester, we observed that 19.3% and 11.8% of non-users started labetalol and nifedipine, respectively, in the 0–3 months postpartum period, compared to 10.4% and 4.0%, respectively, in the prior comparison period.

**Fig 2:**
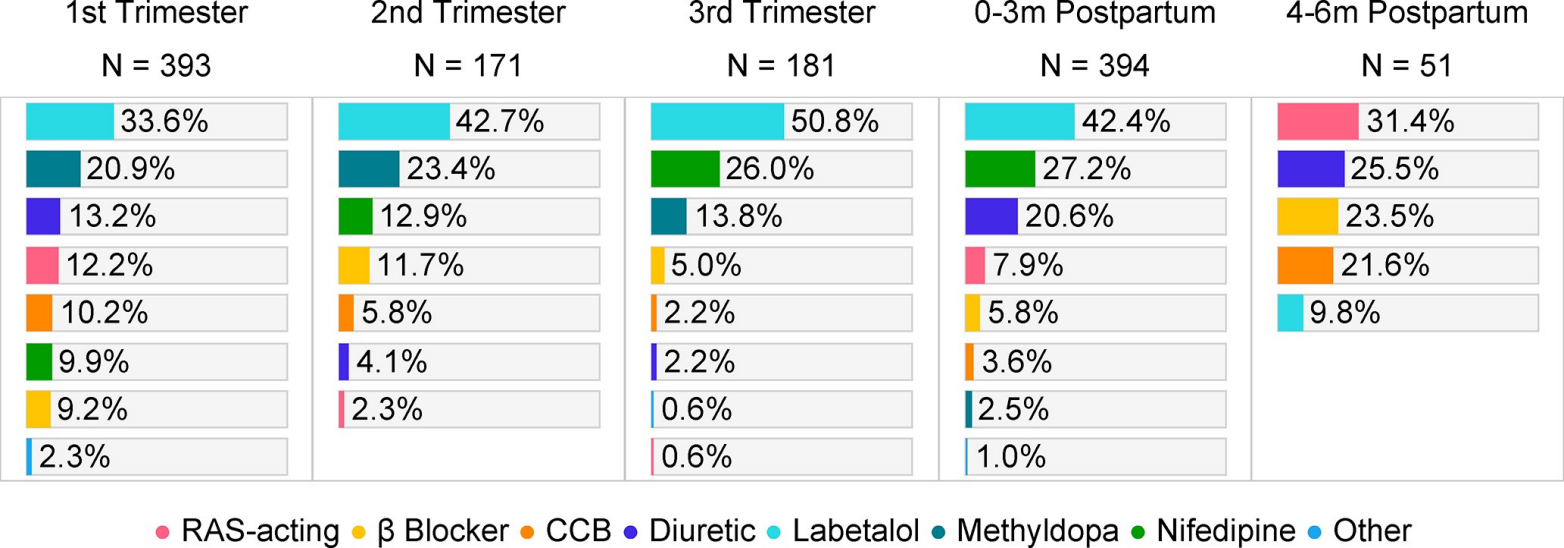
Initial medication choices among initiators by pregnancy-related period, live birth only.

**Fig 3:**
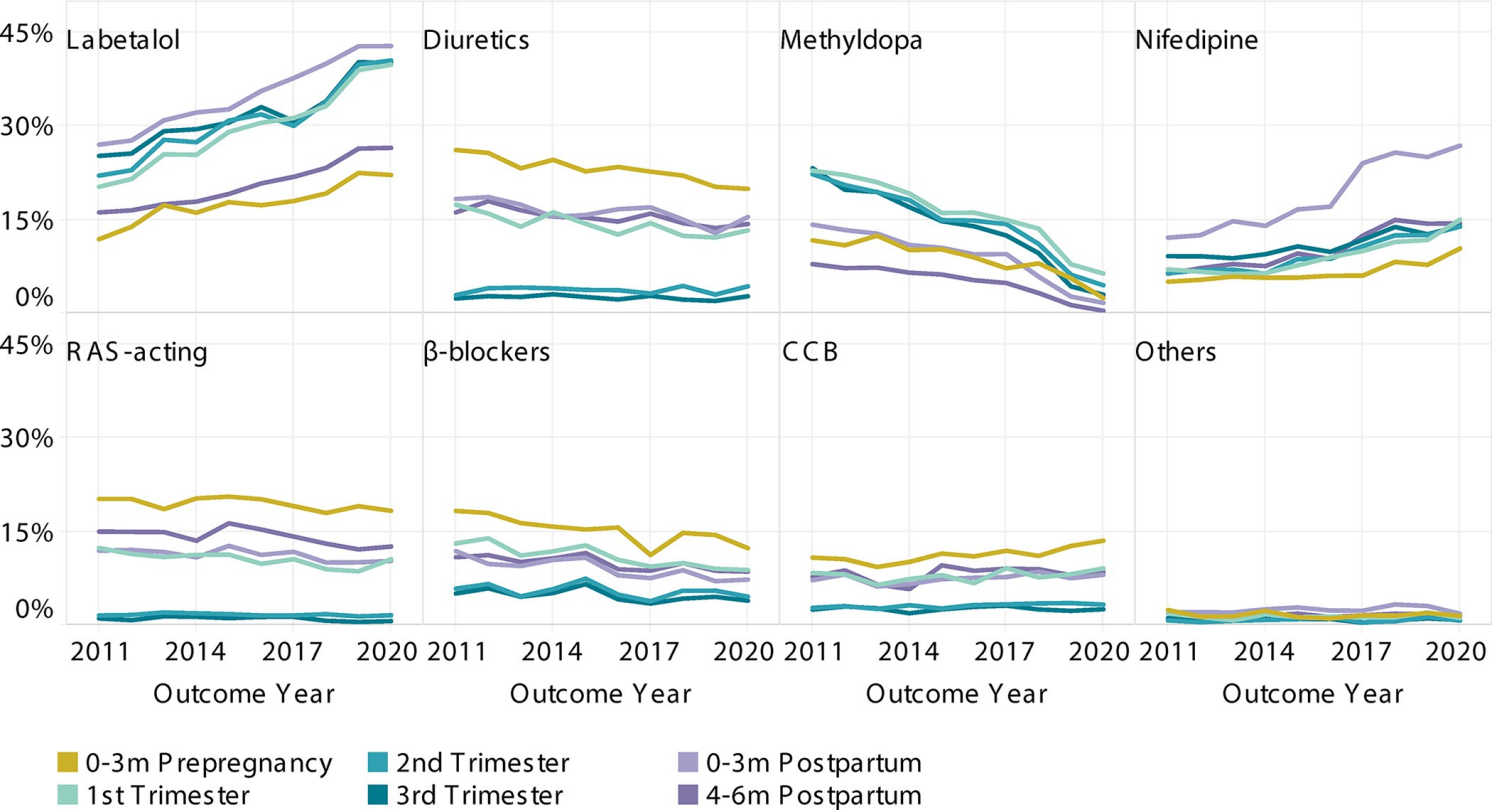
Temporal trends in antihypertensive utilization by pregnancy-related periods, live birth only.

When examining the initial medication choices by pregnancy-related period, we found that three preferred agents—labetalol, nifedipine, and methyldopa—accounted for approximately three-quarters of dispensings among initiators during pregnancy (**Fig 2**). While these three agents remained dominant during pregnancy, we observed different temporal trends in the study period (**Fig 3**). Labetalol and nifedipine became more prevalent compared to methyldopa. Among deliveries in 2011, a similar proportion (~30%) was exposed to labetalol and methyldopa during pregnancy, while in 2020, 52.5% had labetalol, compared to only 7.6% had methyldopa. Additionally, over the decade, the proportion of pregnancies exposed to nifedipine in the first and second trimesters saw a notable increase, jumping from 7% to 14%.

We excluded non-live births from our main analysis of prescribing patterns. However, we observed that pregnancies ending with non-live outcomes were less likely to use or switch to preferred agents in the first trimester compared to live deliveries (**S5 and S6 Tables**).

## Discussion

### Main findings

The main objective of this study was to examine the utilization patterns of antihypertensive medications among pregnant persons with pre-existing hypertension in the US. We found that antihypertensive use declined during pregnancy, especially for RAS-acting agents and diuretics, which pose maternal and fetal risks. We also observed a shift in the preference for antihypertensive agents over time, with labetalol and nifedipine becoming more prevalent than methyldopa, which was historically the first choice. Many patients switched or discontinued their medications during pregnancy, highlighting the need for improved preconception counseling and more evidence to optimize treatment regimes in managing chronic hypertension in this population.

### Increasing prevalence of pre-existing hypertension

Our study provides a comprehensive analysis of antihypertensive utilization patterns among pregnant persons with pre-existing hypertension, spanning the periods before, during, and after pregnancy. The increasing prevalence of pre-existing hypertension in pregnancy over the ten-year study period is consistent with national trends, underscoring the growing public health concern. This rise may be attributed to various factors, including advanced maternal age at conception, lifestyle changes, and the obesity epidemic [20, 21]. The fact that over half of the pregnancies in our cohort occurred in women aged 35 and above further emphasizes the role of maternal age in this trend. Interestingly, the 2017 ACC/AHA guidelines, which redefined hypertension with a BP reading of 130/80 mm HG or higher, introduced new treatment recommendations. Yet, our study didn’t detect a sharp rise in the prevalence of pre-existing chronic hypertension post these guidelines [22, 23]. It’s worth noting that our study’s prevalence of chronic hypertension (0.9% for live births) is notably lower than the national statistics (1.8% during 2011–2020). This difference can be mainly attributed to our study’s focus on hypertension known before pregnancy, excluding those diagnosed early in pregnancy. Furthermore, our findings indicate that 80.4% of pregnancies with pre-existing hypertension had used antihypertensives during the 6-month baseline period. This contrasts with national estimates suggesting that while 4 in 5 women with hypertension are on antihypertensive medications, the age-standardized utilization stands at just 64% [24]. Our specific definition of pre-existing hypertension, which necessitated at least two encounters with hypertension diagnosis codes over a 6-month period [25], might be capturing a patient subset with more comorbidities, or our privately insured population is more likely to afford medication copays.

### Antihypertensive utilization pattern–preferred agents

The dominance of labetalol, nifedipine, and methyldopa in pregnancy aligns with current recommendations. However, the temporal trends—the increasing prevalence of labetalol and nifedipine over methyldopa—suggest a shift in clinical practice. This could be influenced by emerging evidence on the safety and efficacy of these agents or changing perceptions about their risk-benefit. While methyldopa has a long-standing history in pregnancy management and remains the only antihypertensive with an FDA pregnancy category B designation, its preference has eclipsed in recent years [26]. This trend aligns with another recent study conducted over a similar timeframe, which reported infrequent methyldopa use [18]. The 2019 ACOG guideline on chronic hypertension in pregnancy favors labetalol and nifedipine over methyldopa, citing concerns about methyldopa’s effectiveness and potential adverse effects [4, 10]. Furthermore, ACOG advises against using methyldopa postpartum due to its association with depression [27]. Our findings mirror this guidance, showing a decline in methyldopa use postpartum.

### Antihypertensive utilization pattern–concerns with RAS-acting agents

It’s concerning to note the continued use of ACE inhibitors and ARBs during pregnancy, albeit at low rates. Given the consensus against using renin-angiotensin system-acting agents due to potential fetal kidney complications, this finding underscores the need for enhanced preconception counseling and regular medication reviews during pregnancy. The continued use of these agents might reflect gaps in knowledge, challenges in accessing healthcare, or patient-specific considerations that outweigh potential risks. Future studies should delve into understanding these dynamics to ensure safer medication choices during pregnancy.

### Antihypertensive utilization pattern–postpartum use

The dominance of labetalol and nifedipine continued in the 0–3 month postpartum. We found one in six patients with pre-existing hypertension untreated before pregnancy initiated these antihypertensives. This may reflect new-onset preeclampsia or an increased awareness of postpartum hypertension risks. Another study using MarketScan data reported that 64.5% of patients with chronic hypertension sought postpartum care within the first three months. We found that the utilization of frequently used antihypertensive drugs before LMP increased in the 4–6 month postpartum, but not to the same level. This could be due to patients still using medication left from their previous fill, but it may also signify that the transition of care did not happen in time. Future studies are needed to evaluate the quality and outcomes of postpartum care for women with chronic hypertension and identify the barriers and facilitators to optimal care [28].

### Medication transitions: Clinical complexity and future directions

The observed medication switches during pregnancy, especially in the first and second trimesters, highlight the clinical complexities of managing chronic hypertension during this period. These transitions reflect attempts to strike a balance between maternal blood pressure control and fetal safety. They underscore the challenges clinicians face in navigating the risk-benefit tightrope, especially given the limited evidence base for many antihypertensive agents in pregnancy [3]. Patients on combination drugs largely decreased in the second and third trimesters as guidelines recommend monotherapy. However, there has not been sufficient evidence on the net benefit of treatment de-escalation [29]. The CHAP study showed that treating mild chronic hypertension in pregnancy at a blood pressure threshold of 140/90 mm Hg led to fewer adverse maternal and perinatal outcomes compared to waiting for blood pressure to reach 160/105 mm Hg or higher before initiating treatment. Yet, there are still questions not addressed by the CHAP study. For instance, the study included prevalent and new users, showing a more pronounced effect of more aggressive blood pressure control on the former. It suggests that continuing antihypertensive medications yields better outcomes for individuals who were on treatment before pregnancy, while initiating treatment using the same threshold does not produce positive results for those who were not treated before. Moreover, the predominant use of labetalol and nifedipine during the study accounted for over 90% of choices post-randomization compared to less than 50% prior. In a real-world setting, the timing of switching to these two agents may not align with the trial conditions. Additionally, CHAP’s design, which randomized individuals after pregnancy recognition, leaves open the question of whether treatment effects differ based on the timing of antihypertensive initiation. We excluded non-live births from our analysis of prescribing patterns. However, we noted that pregnancies resulting in non-live outcomes were less likely to use or transition to preferred agents during the first trimester compared to live deliveries. Future studies should examine whether suboptimal utilization is associated with the risk of pregnancy losses after balancing the confounding factors. There’s a pressing need for well-designed observational studies with sufficient breadth to evaluate the multitude of possible treatment scenarios considering various maternal risk factors. Such studies can harness real-world data on medication transitions across a broad patient population and diverse clinical scenarios, unrepresented in randomized trials, to provide valuable insights into the safety and efficacy of antihypertensive regimens, guiding clinicians toward more informed decisions.

### Strengths and limitations

To our knowledge, this is the first study examining antihypertensive utilization patterns among pregnant persons with pre-existing hypertension in the US. Our study analyzed utilization patterns before, during, and after pregnancy over a ten-year period from a national sample, providing real-world evidence of changing clinical practices. However, certain limitations might influence the interpretation of our findings. First, the study used administrative claims data, which may not capture all relevant clinical information, such as blood pressure measurements, lifestyle modifications to control hypertension, indications for antihypertensive therapy, and reasons for medication switching or discontinuation. Second, the reliance on administrative claims data might introduce misclassification biases. Drug exposure captured from administrative claims might not mirror actual medication intake. We reported the exposure by requiring a prescription dispensation in the given period and conducted sensitivity analyses considering the days’ supply to provide a range of estimates. Furthermore, it is important to note that the algorithm-based gestational age estimation remains imperfect. However, this limitation will not significantly impact our results as we focused on live-birth pregnancies in our analysis of prescribing patterns. In these cases, the agreement between the predicted gestational age from claims and that obtained from electronic medical records was high (agreement within 7 and 28 days: 82% and 99% for preterm births; 86%-100% for full-term births) [19]. Lastly, the study population does not represent all pregnant persons with pre-existing hypertension in the US, as MarketScan® Commercial Database only included individuals with private health insurance. Future studies should extend to more vulnerable public-insured populations.

## Conclusions

Our study offers a detailed overview of antihypertensive utilization patterns among pregnant persons with pre-existing hypertension in the US. Our findings highlight the decreasing preference for traditionally used agents like methyldopa in favor of labetalol and nifedipine. Importantly, the continued, albeit limited, use of RAS-acting agents during pregnancy calls for heightened preconception counseling and vigilant medication reviews. The observed medication transitions underscore the clinical challenges in managing chronic hypertension during pregnancy, reflecting the ongoing efforts to balance maternal health with fetal safety. In Additionally, we found that one in seven patients discontinued treatment during pregnancy. As the prevalence of chronic hypertension in pregnancy continues to rise, there’s an urgent need for further research to establish the safety and efficacy of various antihypertensive agents. This will ensure optimal maternal and fetal outcomes while addressing the challenges posed by this complex clinical scenario.

## Supporting information

S1 Table Distribution of antihypertensive medications before, during, and after pregnancy, live birth only (N = 12,978). (PDF)

S2 Table Prevalence of antihypertensive medication exposure before, during, and after pregnancy by class considering days’ supply, live birth only (N = 12,978). (PDF)

S3 Table Patterns of antihypertensive exposure from the current to the next pregnancy-related period, live birth only. (PDF)

S4 Table Patterns of antihypertensive exposure from the current to the next pregnancy-related period considering days’ supply, live birth only. (PDF)

S5 Table Prevalence of antihypertensive medication exposure before pregnancy and in the 1st trimester by pregnancy outcome. (PDF)

S6 Table Patterns of antihypertensive exposure from the pre-pregnancy period to the 1^st^ trimester by pregnancy outcome. (PDF)

S1 Appendix Operational definitions of the clinical conditions. (PDF)

S2 Appendix Antihypertensive medications by class. (PDF)
