# Supplementary material for: Antihypertensive utilization patterns among pregnant persons with pre-existing hypertension in the US: A population-based study
Source: PLoS One. 2024 Jul 3;19(7):e0306547. doi: 10.1371/journal.pone.0306547 (PMC11221741; doi:10.1371/journal.pone.0306547)
Supplement: S1 Table — (PDF) [file pone.0306547.s001.pdf]

S1 Table. Distribution of antihypertensive medications before, during, and after pregnancy, live birth only (N = 12,978)

	Pre-pregnancy	1st Trimester	2nd Trimester	3rd Trimester*	0-3m Postpartum	4-6m Postpartum
No Use	3942	4479	5523	5526	4327	5545
Labetalol	2254	3773	3929	4055	4481	2645
Methyldopa	1165	2136	1958	1842	1222	678
Hydrochlorothiazide	2675	1651	401	266	1648	1729
Nifedipine	851	1168	1198	1397	2401	1318
Amlodipine	1275	882	331	277	854	929
Lisinopril	1476	744	108	62	796	955
Metoprolol	1021	758	416	382	690	721
Losartan	535	307	69	39	305	413
Atenolol	351	202	84	68	160	180
Furosemide	116	83	37	31	420	127
Triamterene	272	151	33	21	139	135
Propranolol	201	142	71	57	98	110
Hydralazine	102	97	74	84	218	99
Valsartan	189	105	24	19	113	153
Nebivolol	169	145	37	26	80	110
Carvedilol	134	93	45	35	105	118
Chlorthalidone	161	102	30	19	85	109
Olmesartan	130	88	12	10	87	117
Spironolactone	126	54	14	8	91	117
Clonidine	106	77	28	34	92	54
Diltiazem	93	71	36	29	78	77
Verapamil	89	74	36	31	47	56
Benazepril	106	58	4	4	63	83
Enalapril	54	30	3	0	109	86
Bisoprolol	88	64	15	12	44	46
Acebutolol	41	43	35	29	25	22
Pindolol	19	27	17	13	13	9
Irbesartan	34	19	2	1	14	21
Ramipril	26	15	1	1	15	15
Telmisartan	22	13	1	1	13	17
Guanfacine	11	9	8	7	4	7
Indapamide	21	10	4	2	3	4
Nadolol	13	11	4	4	8	4
Amiloride	8	7	7	6	6	3
Captopril	5	3	1	1	13	10
Prazosin	5	5	2	1	4	12
Torsemide	5	4	2	4	7	6
Doxazosin	3	5	3	0	7	9
Felodipine	4	4	4	3	6	4

	Pre-pregnancy	1st Trimester	2nd Trimester	3rd Trimester*	0-3m Postpartum	4-6m Postpartum
Fosinopril	10	6	1	0	2	2
Quinapril	7	4	0	0	5	5
Metolazone	2	4	2	0	5	1
Azilsartan	1	2	0	1	1	8
Nisoldipine	4	4	1	1	1	1
Aliskiren	4	4	0	0	2	1
Minoxidil	3	2	1	1	2	2
Candesartan	4	2	0	0	1	2
Isradipine	2	2	1	1	1	1
Terazosin	1	0	0	1	3	2
Eplerenone	2	0	1	1	1	1
Trandolapril	1	0	0	0	0	2
Bendroflumethiazide	0	0	0	0	2	0
Timolol	0	0	0	0	1	1
Betaxolol	1	0	0	0	0	0
Nicardipine	0	0	0	0	1	0
Carteolol	0	0	0	0	0	0
Chlorothiazide	0	0	0	0	0	0
Cyclothiazide	0	0	0	0	0	0
Eprosartan	0	0	0	0	0	0
Guanabenz	0	0	0	0	0	0
Levamlodipine	0	0	0	0	0	0
Mecamylamine	0	0	0	0	0	0
Methyclothiazide	0	0	0	0	0	0
Mibefradil	0	0	0	0	0	0
Moexipril	0	0	0	0	0	0
Pargyline	0	0	0	0	0	0
Penbutolol	0	0	0	0	0	0
Perindopril	0	0	0	0	0	0
Polythiazide	0	0	0	0	0	0
Reserpine	0	0	0	0	0	0

*N = 12,881
