# Supplementary material for: Antihypertensive utilization patterns among pregnant persons with pre-existing hypertension in the US: A population-based study
Source: PLoS One. 2024 Jul 3;19(7):e0306547. doi: 10.1371/journal.pone.0306547 (PMC11221741; doi:10.1371/journal.pone.0306547)
Supplement: S2 Table — (PDF) [file pone.0306547.s002.pdf]

S2 Table. Prevalence of antihypertensive medication exposure before, during, and after pregnancy by class considering days' supply, live birth only (N = 12,978)

	**Pre-pregnancy**		**1st Trimester**		**2nd Trimester**		**3rd Trimester***		**0-3m Postpartum**		**4-6m Postpartum**	
	N	%	N	%	N	%	N	%	N	%	N	%
Beta blockers—combined alpha- and beta-receptor	2,652	20.4%	4,130	31.8%	4,345	33.5%	4,529	35.2%	5,447	42.0%	3,397	26.2%
CCB—dihydropyridines	2,417	18.6%	2,376	18.3%	1,828	14.1%	1,881	14.6%	3,509	27.0%	2,632	20.3%
Thiazide or thiazide-type diuretics	3,364	25.9%	2,581	19.9%	815	6.3%	391	3.0%	1,799	13.9%	2,062	15.9%
Central alpha2-agonist and other centrally acting drugs	1,394	10.7%	2,347	18.1%	2,234	17.2%	2,073	16.1%	1,950	15.0%	930	7.2%
Beta blockers—cardioselective	1,665	12.8%	1,384	10.7%	732	5.6%	544	4.2%	987	7.6%	1,053	8.1%
ACE inhibitors	2,046	15.8%	1,405	10.8%	297	2.3%	104	0.8%	1,005	7.7%	1,297	10.0%
ARBs	1,090	8.4%	825	6.4%	192	1.5%	101	0.8%	536	4.1%	785	6.0%
Diuretics—potassium sparing	332	2.6%	238	1.8%	78	0.6%	36	0.3%	153	1.2%	157	1.2%
Diuretics—loop	139	1.1%	117	0.9%	53	0.4%	42	0.3%	436	3.4%	166	1.3%
Beta blockers—noncardioselective	262	2.0%	203	1.6%	106	0.8%	72	0.6%	128	1.0%	133	1.0%
CCB—nondihydropyridines	213	1.6%	182	1.4%	96	0.7%	76	0.6%	138	1.1%	141	1.1%
Direct vasodilators	124	1.0%	117	0.9%	91	0.7%	96	0.7%	249	1.9%	131	1.0%
Beta blockers—cardioselective and vasodilatory	203	1.6%	174	1.3%	62	0.5%	30	0.2%	84	0.6%	126	1.0%
Diuretics—aldosterone antagonists	152	1.2%	97	0.7%	26	0.2%	14	0.1%	94	0.7%	127	1.0%
Beta blockers—intrinsic sympathomimetic activity	71	0.5%	80	0.6%	59	0.5%	51	0.4%	51	0.4%	35	0.3%
Alpha-1 blockers	13	0.1%	12	0.1%	6	0.0%	3	0.0%	14	0.1%	27	0.2%
Direct renin inhibitor	7	0.1%	6	0.0%	0	0.0%	0	0.0%	2	0.0%	1	0.0%
Other Antihypertensives	0	0.0%	0	0.0%	0	0.0%	0	0.0%	0	0.0%	0	0.0%
Any Use	9858	76.0%	9481	73.1%	8202	63.2%	8038	62.4%	9427	72.6%	8236	63.5%
Combination Product	1679	12.9%	1224	9.4%	305	2.4%	139	1.1%	807	6.2%	1041	8.0%

*N = 12,881; ACE: angiotensin-converting enzyme; ARB: angiotensin receptor blocker; CCB: calcium channel blocker
