# Supplementary material for: Antihypertensive utilization patterns among pregnant persons with pre-existing hypertension in the US: A population-based study
Source: PLoS One. 2024 Jul 3;19(7):e0306547. doi: 10.1371/journal.pone.0306547 (PMC11221741; doi:10.1371/journal.pone.0306547)
Supplement: S3 Table — (PDF) [file pone.0306547.s003.pdf]

S3 Table. Patterns of antihypertensive exposure from the current to the next pregnancy-related period, live birth only

		Pre-pregnancy → 1st trimester			1st trimester → 2nd trimester			2nd trimester → 3rd trimester			3rd trimester → 0-3m postpartum			0-3 postpartum → 4-6m postpartum		
Drug Group (C)	Drug Group (N)	N (C)	N (N)	%	N (C)	N (N)	%	N (C)	N (N)	%	N (C)	N (N)	%	N (C)	N (N)	%
RAS-acting	RAS-acting	2,537	1,183	46.6%	1,388	179	12.9%	222	110	49.5%	137	101	73.7%	1,473	1,130	76.7%
	β-blockers	2,537	56	2.2%	1,388	26	1.9%	222	4	1.8%	137	3	2.2%	1,473	26	1.8%
	CCB	2,537	50	2.0%	1,388	9	0.6%	222	3	1.4%	137	1	0.7%	1,473	27	1.8%
	Diuretics	2,537	78	3.1%	1,388	20	1.4%	222	12	5.4%	137	7	5.1%	1,473	62	4.2%
	Labetalol	2,537	674	26.6%	1,388	394	28.4%	222	31	14.0%	137	7	5.1%	1,473	47	3.2%
	Methyldopa	2,537	462	18.2%	1,388	269	19.4%	222	17	7.7%	137	2	1.5%	1,473	9	0.6%
	Nifedipine	2,537	224	8.8%	1,388	128	9.2%	222	17	7.7%	137	6	4.4%	1,473	37	2.5%
	Others	2,537	40	1.6%	1,388	16	1.2%	222	4	1.8%	137	3	2.2%	1,473	10	0.7%
	No Use	2,537	479	18.9%	1,388	389	28.0%	222	42	18.9%	137	23	16.8%	1,473	211	14.3%
β-blockers	RAS-acting	2,006	18	0.9%	1,458	5	0.3%	720	3	0.4%	624	34	5.4%	1,202	25	2.1%
	β-blockers	2,006	1,226	61.1%	1,458	583	40.0%	720	526	73.1%	624	466	74.7%	1,202	892	74.2%
	CCB	2,006	46	2.3%	1,458	19	1.3%	720	11	1.5%	624	25	4.0%	1,202	43	3.6%
	Diuretics	2,006	84	4.2%	1,458	24	1.6%	720	7	1.0%	624	69	11.1%	1,202	72	6.0%
	Labetalol	2,006	346	17.2%	1,458	354	24.3%	720	56	7.8%	624	43	6.9%	1,202	16	1.3%
	Methyldopa	2,006	199	9.9%	1,458	154	10.6%	720	22	3.1%	624	5	0.8%	1,202	9	0.7%
	Nifedipine	2,006	44	2.2%	1,458	53	3.6%	720	28	3.9%	624	30	4.8%	1,202	15	1.2%
	Others	2,006	8	0.4%	1,458	2	0.1%	720	3	0.4%	624	10	1.6%	1,202	2	0.2%
	No Use	2,006	296	14.8%	1,458	312	21.4%	720	101	14.0%	624	81	13.0%	1,202	179	14.9%
CCB	RAS-acting	1,461	19	1.3%							341	11	3.2%	978	33	3.4%
	β-blockers	1,461	29	2.0%	1,033	21	2.0%	408	9	2.2%	341	22	6.5%	978	49	5.0%
	CCB	1,461	798	54.6%	1,033	332	32.1%	408	264	64.7%	341	246	72.1%	978	663	67.8%
	Diuretics	1,461	74	5.1%	1,033	26	2.5%	408	5	1.2%	341	38	11.1%	978	81	8.3%
	Labetalol	1,461	271	18.5%	1,033	262	25.4%	408	58	14.2%	341	31	9.1%	978	24	2.5%
	Methyldopa	1,461	161	11.0%	1,033	133	12.9%	408	11	2.7%	341	1	0.3%	978	14	1.4%
	Nifedipine	1,461	88	6.0%	1,033	78	7.6%	408	10	2.5%	341	12	3.5%	978	9	0.9%
	Others	1,461	9	0.6%	1,033	5	0.5%	408	5	1.2%	341	5	1.5%	978	8	0.8%
	No Use	1,461	218	14.9%	1,033	205	19.8%	408	63	15.4%	341	28	8.2%	978	135	13.8%
Diuretics	RAS-acting	3,024	76	2.5%	1,875	14	0.7%	487	11	2.3%	330	14	4.2%	2,130	138	6.5%
	β-blockers	3,024	81	2.7%	1,875	43	2.3%	487	11	2.3%	330	10	3.0%	2,130	98	4.6%
	CCB	3,024	65	2.1%	1,875	31	1.7%	487	11	2.3%	330	17	5.2%	2,130	94	4.4%
	Diuretics	3,024	1,473	48.7%	1,875	360	19.2%	487	266	54.6%	330	219	66.4%	2,130	1,213	56.9%

		Pre-pregnancy → 1st trimester			1st trimester → 2nd trimester			2nd trimester → 3rd trimester			3rd trimester → 0-3m postpartum			0-3 postpartum → 4-6m postpartum		
Drug Group (C)	Drug Group (N)	N (C)	N (N)	%	N (C)	N (N)	%	N (C)	N (N)	%	N (C)	N (N)	%	N (C)	N (N)	%
	Labetalol	3,024	626	20.7%	1,875	509	27.1%	487	64	13.1%	330	33	10.0%	2,130	137	6.4%
	Methyldopa	3,024	374	12.4%	1,875	307	16.4%	487	27	5.5%	330	6	1.8%	2,130	24	1.1%
	Nifedipine	3,024	165	5.5%	1,875	132	7.0%	487	30	6.2%	330	12	3.6%	2,130	65	3.1%
	Others	3,024	14	0.5%	1,875	13	0.7%	487	4	0.8%	330	7	2.1%	2,130	10	0.5%
	No Use	3,024	531	17.6%	1,875	500	26.7%	487	82	16.8%	330	43	13.0%	2,130	398	18.7%
Labetalol	RAS-acting	2,254	27	1.2%	3,773	8	0.2%	3,929	5	0.1%	4,055	448	11.0%	4,481	491	11.0%
	β-blockers	2,254	14	0.6%	3,773	23	0.6%	3,929	23	0.6%	4,055	248	6.1%	4,481	198	4.4%
	CCB	2,254	23	1.0%	3,773	22	0.6%	3,929	19	0.5%	4,055	269	6.6%	4,481	186	4.2%
	Diuretics	2,254	35	1.6%	3,773	18	0.5%	3,929	17	0.4%	4,055	640	15.8%	4,481	381	8.5%
	Labetalol	2,254	1,764	78.3%	3,773	3,030	80.3%	3,929	3,189	81.2%	4,055	2,866	70.7%	4,481	2,260	50.4%
	Methyldopa	2,254	92	4.1%	3,773	113	3.0%	3,929	58	1.5%	4,055	55	1.4%	4,481	45	1.0%
	Nifedipine	2,254	76	3.4%	3,773	160	4.2%	3,929	179	4.6%	4,055	543	13.4%	4,481	211	4.7%
	Others	2,254	12	0.5%	3,773	17	0.5%	3,929	19	0.5%	4,055	85	2.1%	4,481	37	0.8%
	No Use	2,254	310	13.8%	3,773	494	13.1%	3,929	548	13.9%	4,055	495	12.2%	4,481	1,018	22.7%
Methyldopa	RAS-acting	1,165	9	0.8%	2,136	6	0.3%	1,958	6	0.3%	1,842	314	17.0%	1,222	231	18.9%
	β-blockers	1,165	14	1.2%	2,136	18	0.8%	1,958	15	0.8%	1,842	148	8.0%	1,222	60	4.9%
	CCB	1,165	11	0.9%	2,136	9	0.4%	1,958	8	0.4%	1,842	148	8.0%	1,222	57	4.7%
	Diuretics	1,165	17	1.5%	2,136	7	0.3%	1,958	12	0.6%	1,842	359	19.5%	1,222	117	9.6%
	Labetalol	1,165	130	11.2%	2,136	205	9.6%	1,958	197	10.1%	1,842	292	15.9%	1,222	70	5.7%
	Methyldopa	1,165	906	77.8%	2,136	1,598	74.8%	1,958	1,567	80.0%	1,842	974	52.9%	1,222	567	46.4%
	Nifedipine	1,165	45	3.9%	2,136	110	5.1%	1,958	101	5.2%	1,842	278	15.1%	1,222	61	5.0%
	Others	1,165	7	0.6%	2,136	7	0.3%	1,958	11	0.6%	1,842	50	2.7%	1,222	14	1.1%
	No Use	1,165	116	10.0%	2,136	281	13.2%	1,958	213	10.9%	1,842	220	11.9%	1,222	199	16.3%
Nifedipine	RAS-acting	851	12	1.4%	1,168	5	0.4%	1,198	2	0.2%	1,397	153	11.0%	2,401	291	12.1%
	β-blockers	851	10	1.2%	1,168	11	0.9%	1,198	11	0.9%	1,397	41	2.9%	2,401	81	3.4%
	CCB	851	5	0.6%	1,168	2	0.2%	1,198	2	0.2%	1,397	40	2.9%	2,401	68	2.8%
	Diuretics	851	18	2.1%	1,168	8	0.7%	1,198	5	0.4%	1,397	146	10.5%	2,401	177	7.4%
	Labetalol	851	104	12.2%	1,168	139	11.9%	1,198	108	9.0%	1,397	217	15.5%	2,401	231	9.6%
	Methyldopa	851	65	7.6%	1,168	48	4.1%	1,198	29	2.4%	1,397	43	3.1%	2,401	35	1.5%
	Nifedipine	851	614	72.2%	1,168	854	73.1%	1,198	927	77.4%	1,397	915	65.5%	2,401	1,146	47.7%
	Others	851	10	1.2%	1,168	11	0.9%	1,198	12	1.0%	1,397	26	1.9%	2,401	25	1.0%
	No Use	851	107	12.6%	1,168	139	11.9%	1,198	161	13.4%	1,397	204	14.6%	2,401	552	23.0%

		Pre-pregnancy → 1st trimester			1st trimester → 2nd trimester			2nd trimester → 3rd trimester			3rd trimester → 0-3m postpartum			0-3 postpartum → 4-6m postpartum		
Drug Group (C)	Drug Group (N)	N (C)	N (N)	%	N (C)	N (N)	%	N (C)	N (N)	%	N (C)	N (N)	%	N (C)	N (N)	%
Others	RAS-acting	220	9	4.1%	189	7	3.7%	110	2	1.8%	124	29	23.4%	320	76	23.8%
	β-blockers	220	5	2.3%	189	6	3.2%	110	1	0.9%	124	4	3.2%	320	14	4.4%
	CCB	220	8	3.6%	189	4	2.1%	110	2	1.8%	124	5	4.0%	320	12	3.8%
	Diuretics	220	8	3.6%	189	2	1.1%	110	2	1.8%	124	25	20.2%	320	23	7.2%
	Labetalol	220	49	22.3%	189	45	23.8%	110	8	7.3%	124	22	17.7%	320	34	10.6%
	Methyldopa	220	28	12.7%	189	19	10.1%	110	8	7.3%	124	6	4.8%	320	6	1.9%
	Nifedipine	220	7	3.2%	189	24	12.7%	110	7	6.4%	124	16	12.9%	320	30	9.4%
	Others	220	102	46.4%	189	79	41.8%	110	73	66.4%	124	69	55.6%	320	93	29.1%
	No Use	220	31	14.1%	189	24	12.7%	110	11	10.0%	124	7	5.6%	320	50	15.6%
No Use	RAS-acting	3,942	118	3.0%	4,479	26	0.6%	5,523	7	0.1%	5,526	392	7.1%	4,327	182	4.2%
	β-blockers	3,942	130	3.3%	4,479	80	1.8%	5,523	58	1.1%	5,526	278	5.0%	4,327	130	3.0%
	CCB	3,942	106	2.7%	4,479	29	0.6%	5,523	31	0.6%	5,526	242	4.4%	4,327	100	2.3%
	Diuretics	3,942	163	4.1%	4,479	47	1.0%	5,523	22	0.4%	5,526	634	11.5%	4,327	187	4.3%
	Labetalol	3,942	463	11.7%	4,479	371	8.3%	5,523	573	10.4%	5,526	1,069	19.3%	4,327	193	4.5%
	Methyldopa	3,942	251	6.4%	4,479	153	3.4%	5,523	197	3.6%	5,526	147	2.7%	4,327	57	1.3%
	Nifedipine	3,942	130	3.3%	4,479	104	2.3%	5,523	221	4.0%	5,526	652	11.8%	4,327	66	1.5%
	Others	3,942	23	0.6%	4,479	4	0.1%	5,523	9	0.2%	5,526	67	1.2%	4,327	17	0.4%
	No Use	3,942	2,916	74.0%	4,479	3,771	84.2%	5,523	4,491	81.3%	5,526	3,302	59.8%	4,327	3,604	83.3%
Combination Product	Combination Product	1,400	673	48.1%	809	107	13.2%	143	77	53.8%	97	66	68.0%	787	587	74.6%
	Single Ingredient	1,400	752	53.7%	809	514	63.5%	143	48	33.6%	97	24	24.7%	787	116	14.7%
	No Use	1,400	231	16.5%	809	194	24.0%	143	28	19.6%	97	11	11.3%	787	103	13.1%

C: Current pregnancy-related period; N: Next pregnancy-related period; RAS: renin-angiotensin-system; CCB: calcium channel blocker
