# Supplementary material for: Antihypertensive utilization patterns among pregnant persons with pre-existing hypertension in the US: A population-based study
Source: PLoS One. 2024 Jul 3;19(7):e0306547. doi: 10.1371/journal.pone.0306547 (PMC11221741; doi:10.1371/journal.pone.0306547)
Supplement: S4 Table — (PDF) [file pone.0306547.s004.pdf]

S4 Table. Patterns of antihypertensive exposure from the current to the next pregnancy-related period considering days' supply, live birth only

		**Pre-pregnancy → 1st trimester**			**1st trimester → 2nd trimester**			**2nd trimester → 3rd trimester**			**3rd trimester → 0-3m postpartum**			**0-3 postpartum → 4-6m postpartum**		
Drug Group (C)	Drug Group (N)	N (C)	N (N)	%	N (C)	N (N)	%	N (C)	N (N)	%	N (C)	N (N)	%	N (C)	N (N)	%
RAS-acting	RAS-acting	3,066	2,051	66.9%	2,199	462	21.0%	484	181	37.4%	201	151	75.1%	1,494	1,317	88.2%
	β-blockers	3,066	58	1.9%	2,199	39	1.8%	484	12	2.5%	201	2	1.0%	1,494	16	1.1%
	CCB	3,066	45	1.5%	2,199	19	0.9%	484	7	1.4%	201	4	2.0%	1,494	18	1.2%
	Diuretics	3,066	101	3.3%	2,199	31	1.4%	484	8	1.7%	201	14	7.0%	1,494	40	2.7%
	Labetalol	3,066	802	26.2%	2,199	582	26.5%	484	115	23.8%	201	24	11.9%	1,494	33	2.2%
	Methyldopa	3,066	565	18.4%	2,199	387	17.6%	484	60	12.4%	201	9	4.5%	1,494	10	0.7%
	Nifedipine	3,066	270	8.8%	2,199	230	10.5%	484	45	9.3%	201	15	7.5%	1,494	35	2.3%
	Others	3,066	56	1.8%	2,199	33	1.5%	484	12	2.5%	201	9	4.5%	1,494	10	0.7%
	No Use	3,066	296	9.7%	2,199	514	23.4%	484	78	16.1%	201	13	6.5%	1,494	93	6.2%
β-blockers	RAS-acting	2,308	28	1.2%	1,939	7	0.4%	1,012	2	0.2%	737	33	4.5%	1,340	22	1.6%
	β-blockers	2,308	1,782	77.2%	1,939	926	47.8%	1,012	674	66.6%	737	657	89.1%	1,340	1,098	81.9%
	CCB	2,308	46	2.0%	1,939	28	1.4%	1,012	22	2.2%	737	20	2.7%	1,340	44	3.3%
	Diuretics	2,308	73	3.2%	1,939	45	2.3%	1,012	11	1.1%	737	67	9.1%	1,340	63	4.7%
	Labetalol	2,308	323	14.0%	1,939	436	22.5%	1,012	169	16.7%	737	50	6.8%	1,340	24	1.8%
	Methyldopa	2,308	169	7.3%	1,939	197	10.2%	1,012	42	4.2%	737	7	0.9%	1,340	8	0.6%
	Nifedipine	2,308	40	1.7%	1,939	54	2.8%	1,012	39	3.9%	737	28	3.8%	1,340	9	0.7%
	Others	2,308	9	0.4%				1,012	5	0.5%	737	8	1.1%	1,340	2	0.1%
	No Use	2,308	186	8.1%	1,939	322	16.6%	1,012	83	8.2%	737	31	4.2%	1,340	115	8.6%
CCB	RAS-acting	1,710	21	1.2%	1,417	6	0.4%				424	11	2.6%	1,044	23	2.2%
	β-blockers	1,710	38	2.2%	1,417	32	2.3%	623	8	1.3%	424	23	5.4%	1,044	42	4.0%
	CCB	1,710	1,235	72.2%	1,417	554	39.1%	623	361	57.9%	424	353	83.3%	1,044	795	76.1%
	Diuretics	1,710	85	5.0%	1,417	43	3.0%	623	12	1.9%	424	38	9.0%	1,044	76	7.3%
	Labetalol	1,710	242	14.2%	1,417	354	25.0%	623	110	17.7%	424	29	6.8%	1,044	31	3.0%
	Methyldopa	1,710	141	8.2%	1,417	168	11.9%	623	33	5.3%	424	4	0.9%	1,044	10	1.0%
	Nifedipine	1,710	83	4.9%	1,417	80	5.6%	623	38	6.1%	424	15	3.5%	1,044	12	1.1%
	Others	1,710	8	0.5%	1,417	4	0.3%	623	3	0.5%	424	6	1.4%	1,044	7	0.7%
	No Use	1,710	116	6.8%	1,417	224	15.8%	623	71	11.4%	424	20	4.7%	1,044	76	7.3%
Diuretics	RAS-acting	3,558	83	2.3%	2,744	20	0.7%	884	11	1.2%	445	16	3.6%	2,205	108	4.9%
	β-blockers	3,558	69	1.9%	2,744	53	1.9%	884	19	2.1%	445	18	4.0%	2,205	90	4.1%
	CCB	3,558	63	1.8%	2,744	45	1.6%	884	17	1.9%	445	23	5.2%	2,205	92	4.2%
	Diuretics	3,558	2,428	68.2%	2,744	762	27.8%	884	386	43.7%	445	333	74.8%	2,205	1,464	66.4%

		Pre-pregnancy → 1st trimester			1st trimester → 2nd trimester			2nd trimester → 3rd trimester			3rd trimester → 0-3m postpartum			0-3 postpartum → 4-6m postpartum		
Drug Group (C)	Drug Group (N)	N (C)	N (N)	%	N (C)	N (N)	%	N (C)	N (N)	%	N (C)	N (N)	%	N (C)	N (N)	%
	Labetalol	3,558	624	17.5%	2,744	703	25.6%	884	203	23.0%	445	40	9.0%	2,205	140	6.3%
	Methyldopa	3,558	365	10.3%	2,744	399	14.5%	884	95	10.7%	445	10	2.2%	2,205	27	1.2%
	Nifedipine	3,558	151	4.2%	2,744	200	7.3%	884	52	5.9%	445	14	3.1%	2,205	63	2.9%
	Others	3,558	19	0.5%	2,744	8	0.3%	884	7	0.8%	445	8	1.8%	2,205	10	0.5%
	No Use	3,558	326	9.2%	2,744	615	22.4%	884	117	13.2%	445	19	4.3%	2,205	245	11.1%
Labetalol	RAS-acting	2,512	33	1.3%	4,038	16	0.4%	4,296	6	0.1%	4,492	454	10.1%	5,370	635	11.8%
	β-blockers	2,512	20	0.8%	4,038	24	0.6%	4,296	21	0.5%	4,492	265	5.9%	5,370	236	4.4%
	CCB	2,512	22	0.9%	4,038	16	0.4%	4,296	18	0.4%	4,492	260	5.8%	5,370	223	4.2%
	Diuretics	2,512	47	1.9%	4,038	17	0.4%	4,296	12	0.3%	4,492	679	15.1%	5,370	458	8.5%
	Labetalol	2,512	2,129	84.8%	4,038	3,467	85.9%	4,296	3,726	86.7%	4,492	3,914	87.1%	5,370	2,991	55.7%
	Methyldopa	2,512	101	4.0%	4,038	135	3.3%	4,296	78	1.8%	4,492	52	1.2%	5,370	50	0.9%
	Nifedipine	2,512	76	3.0%	4,038	158	3.9%	4,296	201	4.7%	4,492	531	11.8%	5,370	313	5.8%
	Others	2,512	10	0.4%	4,038	14	0.3%	4,296	20	0.5%	4,492	80	1.8%	5,370	50	0.9%
	No Use	2,512	167	6.6%	4,038	281	7.0%	4,296	324	7.5%	4,492	188	4.2%	5,370	892	16.6%
Methyldopa	RAS-acting	1,269	17	1.3%	2,260	13	0.6%	2,191	8	0.4%	2,035	331	16.3%	1,857	376	20.2%
	β-blockers	1,269	15	1.2%	2,260	16	0.7%	2,191	10	0.5%	2,035	154	7.6%	1,857	100	5.4%
	CCB	1,269	9	0.7%	2,260	9	0.4%	2,191	5	0.2%	2,035	152	7.5%	1,857	85	4.6%
	Diuretics	1,269	18	1.4%	2,260	6	0.3%	2,191	9	0.4%	2,035	370	18.2%	1,857	180	9.7%
	Labetalol	1,269	144	11.3%	2,260	171	7.6%	2,191	237	10.8%	2,035	350	17.2%	1,857	130	7.0%
	Methyldopa	1,269	1,054	83.1%	2,260	1,839	81.4%	2,191	1,802	82.2%	2,035	1,628	80.0%	1,857	785	42.3%
	Nifedipine	1,269	49	3.9%	2,260	118	5.2%	2,191	132	6.0%	2,035	293	14.4%	1,857	148	8.0%
	Others	1,269	5	0.4%	2,260	9	0.4%	2,191	14	0.6%	2,035	49	2.4%	1,857	24	1.3%
	No Use	1,269	60	4.7%	2,260	177	7.8%	2,191	157	7.2%	2,035	92	4.5%	1,857	248	13.4%
Nifedipine	RAS-acting	963	16	1.7%	1,283	11	0.9%	1,352	1	0.1%	1,546	165	10.7%	2,741	374	13.6%
	β-blockers	963	14	1.5%	1,283	3	0.2%	1,352	7	0.5%	1,546	28	1.8%	2,741	69	2.5%
	CCB	963	5	0.5%	1,283	5	0.4%	1,352	2	0.1%	1,546	43	2.8%	2,741	64	2.3%
	Diuretics	963	18	1.9%	1,283	6	0.5%	1,352	6	0.4%	1,546	150	9.7%	2,741	184	6.7%
	Labetalol	963	130	13.5%	1,283	118	9.2%	1,352	118	8.7%	1,546	218	14.1%	2,741	280	10.2%
	Methyldopa	963	63	6.5%	1,283	59	4.6%	1,352	38	2.8%	1,546	65	4.2%	2,741	47	1.7%
	Nifedipine	963	747	77.6%	1,283	1,026	80.0%	1,352	1,120	82.8%	1,546	1,326	85.8%	2,741	1,483	54.1%
	Others	963	14	1.5%	1,283	13	1.0%	1,352	14	1.0%	1,546	36	2.3%	2,741	39	1.4%
	No Use	963	63	6.5%	1,283	88	6.9%	1,352	91	6.7%	1,546	79	5.1%	2,741	464	16.9%

		**Pre-pregnancy → 1st trimester**			**1st trimester → 2nd trimester**			**2nd trimester → 3rd trimester**			**3rd trimester → 0-3m postpartum**			**0-3 postpartum → 4-6m postpartum**		
Drug Group (C)	Drug Group (N)	N (C)	N (N)	%	N (C)	N (N)	%	N (C)	N (N)	%	N (C)	N (N)	%	N (C)	N (N)	%
Others	RAS-acting	260	15	5.8%	225	9	4.0%	146	2	1.4%	142	34	23.9%	360	102	28.3%
	β-blockers	260	6	2.3%	225	4	1.8%	146	2	1.4%	142	5	3.5%	360	4	1.1%
	CCB	260	6	2.3%	225	4	1.8%	146	3	2.1%	142	6	4.2%	360	9	2.5%
	Diuretics	260	7	2.7%	225	4	1.8%				142	22	15.5%	360	25	6.9%
	Labetalol	260	47	18.1%	225	44	19.6%	146	16	11.0%	142	27	19.0%	360	33	9.2%
	Methyldopa	260	32	12.3%	225	15	6.7%	146	7	4.8%	142	11	7.7%	360	7	1.9%
	Nifedipine	260	17	6.5%	225	24	10.7%	146	15	10.3%	142	23	16.2%	360	30	8.3%
	Others	260	138	53.1%	225	115	51.1%	146	90	61.6%	142	112	78.9%	360	130	36.1%
	No Use	260	21	8.1%	225	24	10.7%	146	11	7.5%	142	1	0.7%	360	42	11.7%
No Use	RAS-acting	3,120	67	2.1%	3,497	8	0.2%	4,776	5	0.1%	4,843	308	6.4%	3,551	121	3.4%
	β-blockers	3,120	60	1.9%	3,497	36	1.0%	4,776	31	0.6%	4,843	201	4.2%	3,551	69	1.9%
	CCB	3,120	62	2.0%	3,497	15	0.4%	4,776	18	0.4%	4,843	187	3.9%	3,551	69	1.9%
	Diuretics	3,120	73	2.3%	3,497	15	0.4%	4,776	13	0.3%	4,843	520	10.7%	3,551	121	3.4%
	Labetalol	3,120	246	7.9%	3,497	186	5.3%	4,776	394	8.2%	4,843	795	16.4%	3,551	70	2.0%
	Methyldopa	3,120	157	5.0%	3,497	92	2.6%	4,776	144	3.0%	4,843	86	1.8%	3,551	25	0.7%
	Nifedipine	3,120	74	2.4%	3,497	56	1.6%	4,776	164	3.4%	4,843	517	10.7%	3,551	18	0.5%
	Others	3,120	15	0.5%	3,497	1	0.0%	4,776	6	0.1%	4,843	51	1.1%	3,551	8	0.2%
	No Use	3,120	2,544	81.5%	3,497	3,126	89.4%	4,776	4,056	84.9%	4,843	3,123	64.5%	3,551	3,164	89.1%
Combination Product	Combination Product	1,679	1,126	67.1%	1,224	287	23.4%	305	126	41.3%	139	100	71.9%	807	692	85.7%
	Single Ingredient	1,679	811	48.3%	1,224	702	57.4%	305	146	47.9%	139	40	28.8%	807	77	9.5%
	No Use	1,679	132	7.9%	1,224	251	20.5%	305	45	14.8%	139	6	4.3%	807	48	5.9%

C: Current pregnancy-related period; N: Next pregnancy-related period; RAS: renin-angiotensin-system; CCB: calcium channel blocker
