# Supplementary material for: Antihypertensive utilization patterns among pregnant persons with pre-existing hypertension in the US: A population-based study
Source: PLoS One. 2024 Jul 3;19(7):e0306547. doi: 10.1371/journal.pone.0306547 (PMC11221741; doi:10.1371/journal.pone.0306547)
Supplement: S5 Table — (PDF) [file pone.0306547.s005.pdf]

S5 Table. Prevalence of antihypertensive medication exposure before pregnancy and in the 1st trimester by pregnancy outcome

	**Pre-pregnancy**		**1st trimester**	
	**N**	**%**	**N**	**%**
**Live births (N = 12,978)**				
RAS-acting	2,537	19.5%	1,388	10.7%
β-blockers	2,006	15.5%	1,458	11.2%
CCB	1,461	11.3%	1,033	8.0%
Diuretics	3,024	23.3%	1,875	14.4%
Labetalol	2,254	17.4%	3,773	29.1%
Methyldopa	1,165	9.0%	2,136	16.5%
Nifedipine	851	6.6%	1,168	9.0%
Others	220	1.7%	189	1.5%
No Use	3,942	30.4%	4,479	34.5%
**Non-live births (N = 7,598)**				
RAS-acting	1,875	24.7%	1,284	16.9%
β-blockers	1,209	15.9%	923	12.1%
CCB	1,103	14.5%	801	10.5%
Diuretics	2,061	27.1%	1,426	18.8%
Labetalol	1,119	14.7%	1,515	19.9%
Methyldopa	547	7.2%	807	10.6%
Nifedipine	446	5.9%	470	6.2%
Others	162	2.1%	126	1.7%
No Use	2,214	29.1%	2,898	38.1%

RAS: renin-angiotensin-system; CCB: calcium channel blocker
