# Supplementary material for: Antihypertensive utilization patterns among pregnant persons with pre-existing hypertension in the US: A population-based study
Source: PLoS One. 2024 Jul 3;19(7):e0306547. doi: 10.1371/journal.pone.0306547 (PMC11221741; doi:10.1371/journal.pone.0306547)
Supplement: S6 Table — (PDF) [file pone.0306547.s006.pdf]

S6 Table. Patterns of antihypertensive exposure from the pre-pregnancy period to the 1st trimester by pregnancy outcome

		Pre-pregnancy → 1st trimester					
		Live births (N = 12,978)			Non-live births (N = 7,598)		
Drug Group (C)	Drug Group (N)	N (Current)	N (Next)	%	N (Current)	N (Next)	%
RAS-acting	RAS-acting	2,537	1,183	46.6%	1,875	1,097	58.5%
	β-blockers	2,537	56	2.2%	1,875	44	2.3%
	CCB	2,537	50	2.0%	1,875	43	2.3%
	Diuretics	2,537	78	3.1%	1,875	78	4.2%
	Labetalol	2,537	674	26.6%	1,875	291	15.5%
	Methyldopa	2,537	462	18.2%	1,875	205	10.9%
	Nifedipine	2,537	224	8.8%	1,875	74	3.9%
	Other	2,537	40	1.6%	1,875	32	1.7%
	No Use	2,537	479	18.9%	1,875	367	19.6%
β-blockers	RAS-acting	2,006	18	0.9%	1,209	28	2.3%
	β-blockers	2,006	1,226	61.1%	1,209	777	64.3%
	CCB	2,006	46	2.3%	1,209	42	3.5%
	Diuretics	2,006	84	4.2%	1,209	63	5.2%
	Labetalol	2,006	346	17.2%	1,209	114	9.4%
	Methyldopa	2,006	199	9.9%	1,209	57	4.7%
	Nifedipine	2,006	44	2.2%	1,209	11	0.9%
	Other	2,006	8	0.4%	1,209	6	0.5%
	No Use	2,006	296	14.8%	1,209	203	16.8%
CCB	RAS-acting	1,461	19	1.3%	1,103	20	1.8%
	β-blockers	1,461	29	2.0%	1,103	35	3.2%
	CCB	1,461	798	54.6%	1,103	631	57.2%
	Diuretics	1,461	74	5.1%	1,103	58	5.3%
	Labetalol	1,461	271	18.5%	1,103	135	12.2%
	Methyldopa	1,461	161	11.0%	1,103	67	6.1%
	Nifedipine	1,461	88	6.0%	1,103	30	2.7%
	Other	1,461	9	0.6%	1,103	6	0.5%
	No Use	1,461	218	14.9%	1,103	206	18.7%
Diuretics	RAS-acting	3,024	76	2.5%	2,061	82	4.0%
	β-blockers	3,024	81	2.7%	2,061	62	3.0%
	CCB	3,024	65	2.1%	2,061	71	3.4%
	Diuretics	3,024	1,473	48.7%	2,061	1,107	53.7%
	Labetalol	3,024	626	20.7%	2,061	231	11.2%
	Methyldopa	3,024	374	12.4%	2,061	137	6.6%
	Nifedipine	3,024	165	5.5%	2,061	51	2.5%
	Other	3,024	14	0.5%	2,061	12	0.6%
	No Use	3,024	531	17.6%	2,061	430	20.9%
Labetalol	RAS-acting	2,254	27	1.2%	1,119	21	1.9%
	β-blockers	2,254	14	0.6%	1,119	8	0.7%
	CCB	2,254	23	1.0%	1,119	13	1.2%
	Diuretics	2,254	35	1.6%	1,119	27	2.4%
	Labetalol	2,254	1,764	78.3%	1,119	770	68.8%

		Pre-pregnancy → 1st trimester					
		**Live births (N = 12,978)**			**Non-live births (N = 7,598)**		
	Methyldopa	2,254	92	4.1%	1,119	40	3.6%
	Nifedipine	2,254	76	3.4%	1,119	36	3.2%
	Other	2,254	12	0.5%	1,119	7	0.6%
	No Use	2,254	310	13.8%	1,119	237	21.2%
Methyldopa	RAS-acting	1,165	9	0.8%	547	15	2.7%
	β-blockers	1,165	14	1.2%	547	7	1.3%
	CCB	1,165	11	0.9%	547	3	0.5%
	Diuretics	1,165	17	1.5%	547	18	3.3%
	Labetalol	1,165	130	11.2%	547	47	8.6%
	Methyldopa	1,165	906	77.8%	547	365	66.7%
	Nifedipine	1,165	45	3.9%	547	21	3.8%
	Other	1,165	7	0.6%	547	3	0.5%
	No Use	1,165	116	10.0%	547	94	17.2%
Nifedipine	RAS-acting	851	12	1.4%	446	14	3.1%
	β-blockers	851	10	1.2%	446	8	1.8%
	CCB	851	5	0.6%	446	5	1.1%
	Diuretics	851	18	2.1%	446	8	1.8%
	Labetalol	851	104	12.2%	446	38	8.5%
	Methyldopa	851	65	7.6%	446	25	5.6%
	Nifedipine	851	614	72.2%	446	278	62.3%
	Other	851	10	1.2%	446	7	1.6%
	No Use	851	107	12.6%	446	83	18.6%
Other	RAS-acting	220	9	4.1%	162	23	14.2%
	β-blockers	220	5	2.3%	162	8	4.9%
	CCB	220	8	3.6%	162	5	3.1%
	Diuretics	220	8	3.6%	162	10	6.2%
	Labetalol	220	49	22.3%	162	19	11.7%
	Methyldopa	220	28	12.7%	162	16	9.9%
	Nifedipine	220	7	3.2%	162	5	3.1%
	Other	220	102	46.4%	162	74	45.7%
	No Use	220	31	14.1%	162	23	14.2%
No Use	RAS-acting	3,942	118	3.0%	2,214	118	5.3%
	β-blockers	3,942	130	3.3%	2,214	79	3.6%
	CCB	3,942	106	2.7%	2,214	66	3.0%
	Diuretics	3,942	163	4.1%	2,214	121	5.5%
	Labetalol	3,942	463	11.7%	2,214	157	7.1%
	Methyldopa	3,942	251	6.4%	2,214	79	3.6%
	Nifedipine	3,942	130	3.3%	2,214	48	2.2%
	Other	3,942	23	0.6%	2,214	4	0.2%
	No Use	3,942	2,916	74.0%	2,214	1,732	78.2%

RAS: renin-angiotensin-system; CCB: calcium channel blocker
