# Supplementary material for: Antihypertensive utilization patterns among pregnant persons with pre-existing hypertension in the US: A population-based study
Source: PLoS One. 2024 Jul 3;19(7):e0306547. doi: 10.1371/journal.pone.0306547 (PMC11221741; doi:10.1371/journal.pone.0306547)
Supplement: S1 Appendix — (PDF) [file pone.0306547.s007.pdf]

S1 Appendix. Operational definitions of the clinical conditions

**Characteristics**	**Operational Definition**
Essential Hypertension	**ICD-9-CM:** 401x<br>**ICD-10-CM:** I10x
Preeclampsia<br>(from 20 weeks of gestation to 12 weeks postpartum)	**ICD-9-CM:** 6424x, 6425x, 6426x, 6427x<br>**ICD-10-CM:** O14x, O15x
Disorders of lipid metabolism	**CCS:** 53
Diabetes	**CCS:** 49, 50
Anxiety	**CCS:** 651
Depression	**ICD-9-CM:** 29620, 29621, 29622, 29623, 29624, 29625, 29626, 29630, 29631, 29632, 29633, 29634, 29635, 29636, 3004, 311<br>**ICD-10-CM:** F320, F321, F322, F323, F324, F325, F329, F330, F331, F332, F333, F3340, F3341, F3342, F339, F341, F3281, F3289
Chronic Kidney Disease	**CCS:** 158
Ischemic Heart Disease	**ICD-9-CM:** 41000, 41001, 41002, 41010, 41011, 41012, 41020, 41021, 41022, 41030, 41031, 41032, 41040, 41041, 41042, 41050, 41051, 41052, 41060, 41061, 41062, 41070, 41071, 41072, 41080, 41081, 41082, 41090, 41091, 41092, 4110, 4111, 41181, 41189, 412, 4130, 4131, 4139, 41400, 41401, 41402, 41403, 41404, 41405, 41406, 41407, 41412, 4142, 4143, 4144, 4148, 4149<br>**ICD-10-CM:** I200, I201, I208, I209, I2101, I2102, I2109, I2111, I2119, I2121, I2129, I213, I214, I220, I221, I222, I228, I229, I240, I241, I248, I249, I2510, I25110, I25111, I25118, I25119, I252, I2542, I255, I256, I25700, I25701, I25708, I25709, I25710, I25711, I25718, I25719, I25720, I25721, I25728, I25729, I25730, I25731, I25738, I25739, I25750, I25751, I25758, I25759, I25760, I25761, I25768, I25769, I25790, I25791, I25798, I25799, I25810, I25811, I25812, I2582, I2583, I2584, I2589, I259
Congestion Heart Failure	**ICD-9-CM:** 39891, 40201, 40211, 40291, 40401, 40403, 40411, 40413, 40491, 40493, 4280, 4281, 42820, 42821, 42822, 42823, 42830, 42831, 42832, 42833, 42840, 42841, 42842, 42843, 4289<br>**ICD-10-CM:** I0981, I110, I130, I132, I501, I5020, I5021, I5022, I5023, I5030, I5031, I5032, I5033, I5040, I5041, I5042, I5043, I509
1st trimester Prenatal Care	**[Practitioner Type] AND ([Prenatal Ultrasound] OR [Obstetric Panel] OR [Stand Alone Prenatal Visits] OR [Pregnancy Diagnosis])**
Practitioner Type	Medical Doctor, Osteopathic Medicine, Internal Medicine, Family Practice, Obstetrics & Gynecology, Midwife, Nurse Practitioner, Physician Assistant
Prenatal Ultrasound	**CPT:** 76801, 76805, 76811, 76813, 76815, 76816, 76817, 76818, 76819, 76820, 76821, 76825, 76826, 76827, 76828<br>**ICD-9-CM Procedure:** 8878<br>**ICD-10-PCS:** BY49ZZZ, BY4BZZZ, BY4CZZZ, BY4DZZZ, BY4FZZZ, BY4GZZZ
Obstetric Panel	**CPT:** 80055, 80081
Stand Alone Prenatal Visits	**CPT/HCPCS:** 99500, 0500F, 0501F, 0502F, H1000, H1001, H1002, H1003, H1004
Pregnancy Diagnosis	**ICD-9-CM:** 64003, 64083, 64093, 64103, 64113, 64123, 64133, 64183, 64193, 64203, 64213, 64223, 64233, 64243, 64253, 64263, 64273, 64293, 64303, 64313, 64323, 64383, 64393, 64403, 64413, 64513, 64523, 64603, 64613, 64623, 64633, 64643, 64653, 64663, 64673, 64683, 64693, 64703, 64713, 64723, 64733, 64743, 64753, 64763, 64783, 64793, 64803, 64813, 64823, 64833, 64843, 64853, 64863, 64873, 64883, 64893, 64903, 64913, 64923, 64933, 64943, 64953, 64963, 64973, 65103, 65113, 65123, 65133, 65143, 65153, 65163, 65173, 65183, 65193, 65203, 65213, 65223, 65233, 65243, 65253, 65263, 65273, 65283, 65293, 65303, 65313, 65323, 65333, 65343, 65353, 65363, 65373, 65383, 65393, 65403, 65413, 65423, 65433, 65443, 65453, 65463, 65473, 65483, 65493, 65503, 65513, 65523, 65533, 65543, 65553, 65563, 65573, 65583, 65593, 65603, 65613, 65623, 65633, 65643, 65653, 65663, 65673, 65683, 65693, 65703, 65803, 65813, 65823, 65833, 65843, 65883, 65893, 65903, 65913, 65923, 65933, 65943, 65953, 65963, 65973, 65983, 65993, 67803, 67813, 67903, 67913, V220, V221, V222, V230, V231, V232, V233, V2341, V2342, V2349, V235, V237, V2381, V2382, V2383, V2384, V2385, V2386, V2387, V2389, V239, V280, V281, V282, V283, V284, V285, V286, V2881, V2882, V2889, V289

	**ICD-10-CM:** O0900, O0901, O0902, O0903, O0910, O0911, O0912, O0913, O09211, O09212, O09213, O09219, O09291, O09292, O09293, O09299, O0930, O0931, O0932, O0933, O0940, O0941, O0942, O0943, O09511, O09512, O09513, O09519, O09521, O09522, O09523, O09529, O09611, O09612, O09613, O09619, O09621, O09622, O09623, O09629, O0970, O0971, O0972, O0973, O09811, O09812, O09813, O09819, O09821, O09822, O09823, O09829, O09891, O09892, O09893, O09899, O0990, O0991, O0992, O0993, O09A0, O09A1, O09A2, O09A3, O10011, O10012, O10013, O10019, O10111, O10112, O10113, O10119, O10211, O10212, O10213, O10219, O10311, O10312, O10313, O10319, O10411, O10412, O10413, O10419, O10911, O10912, O10913, O10919, O111, O112, O113, O119, O1200, O1201, O1202, O1203, O1210, O1211, O1212, O1213, O131, O132, O133, O139, O1400, O1402, O1403, O1410, O1412, O1413, O1420, O1422, O1423, O1490, O1492, O1493, O1500, O1502, O1503, O151, O159, O161, O162, O163, O169, O200, O208, O209, O210, O211, O212, O218, O219, O2200, O2201, O2202, O2203, O2210, O2211, O2212, O2213, O2220, O2221, O2222, O2223, O2230, O2231, O2232, O2233, O2240, O2241, O2242, O2243, O2250, O2251, O2252, O2253, O2281, O2282, O2283, O2289, O2290, O2291, O2292, O2293, O2300, O2301, O2302, O2303, O2310, O2311, O2312, O2313, O2320, O2321, O2322, O2323, O2330, O2331, O2332, O2333, O2340, O2341, O2342, O2343, O23511, O23512, O23513, O23519, O23521, O23522, O23523, O23529, O23591, O23592, O23593, O23599, O2390, O2391, O2392, O2393, O24011, O24012, O24013, O24019, O24111, O24112, O24113, O24119, O24311, O24312, O24313, O24319, O24410, O24414, O24415, O24419, O24811, O24812, O24813, O24819, O24911, O24912, O24913, O24919, O2510, O2511, O2512, O2513, O2600, O2601, O2602, O2603, O2610, O2611, O2612, O2613, O2620, O2621, O2622, O2623, O2630, O2631, O2632, O2633, O2640, O2641, O2642, O2643, O2650, O2651, O2652, O2653, O26611, O26612, O26613, O26619, O266711, O266712, O266713, O266719, O266811, O266812, O266813, O266819

ICD-9/10-CM: International Classification of Diseases, Ninth/Tenth Revision, Clinical Modification
ICD-10-PCS: International Classification of Diseases, Ninth/Tenth Revision, Procedure Coding System
CPT: Current Procedural Terminology
HCPCS: Healthcare Common Procedure Coding System
CCS: Clinical Classifications Software for ICD-9-CM and ICD-10-CM
