# Supplementary material for: Antihypertensive utilization patterns among pregnant persons with pre-existing hypertension in the US: A population-based study
Source: PLoS One. 2024 Jul 3;19(7):e0306547. doi: 10.1371/journal.pone.0306547 (PMC11221741; doi:10.1371/journal.pone.0306547)
Supplement: S2 Appendix — (PDF) [file pone.0306547.s008.pdf]

S2 Appendix. Antihypertensive Medications by Class

Drug Groups in Medication Transition Analysis	Drug Class	Drug
Beta blockers	Beta blockers—cardioselective	Atenolol
		Betaxolol
		Bisoprolol
		Metoprolol
	Beta blockers—cardioselective and vasodilatory	Nebivolol
	Beta blockers—combined alpha- and beta-receptor	Carvedilol
	Beta blockers—intrinsic sympathomimetic activity	Acebutolol
		Penbutolol
		Pindolol
	Beta blockers—noncardioselective	Carteolol
		Nadolol
		Propranolol
		Timolol
CCB	CCB—dihydropyridines	Amlodipine
		Felodipine
		Isradipine
		Levamlodipine
		Nicardipine
		Nisoldipine
	CCB—nondihydropyridines	Diltiazem
		Mibefradil
		Verapamil
Diuretics	Diuretics—aldosterone antagonists	Eplerenone
		Spironolactone
	Diuretics—loop	Furosemide
		Torsemide
	Diuretics—potassium sparing	Amiloride
		Triamterene
	Thiazide or thiazide-type diuretics	Bendroflumethiazide
		Chlorothiazide
		Chlorthalidone
		Cyclothiazide
		Hydrochlorothiazide
		Indapamide
		Methyclothiazide
		Metolazone
		Polythiazide
RAS-acting agents	ACE inhibitors	Benazepril
		Captopril
		Enalapril
		Fosinopril
		Lisinopril
		Moexipril
		Perindopril
		Quinapril

Drug Groups in Medication Transition Analysis	Drug Class	Drug
		Ramipril
		Trandolapril
	ARBs	Azilsartan
		Candesartan
		Eprosartan
		Irbesartan
		Losartan
		Olmesartan
		Telmisartan
		Valsartan
	Direct renin inhibitor	Aliskiren
Labetalol	Beta blockers—combined alpha- and beta-receptor	Labetalol
Methyldopa	Central alpha2-agonist and other centrally acting drugs	Methyldopa
Nifedipine	CCB—dihydropyridines	Nifedipine
Others	Alpha-1 blockers	Doxazosin
		Prazosin
		Terazosin
	Central alpha2-agonist and other centrally acting drugs	Clonidine
		Guanabenz
		Guanfacine
		Reserpine
	Direct vasodilators	Hydralazine
		Minoxidil
	Other Antihypertensives	Mecamylamine
		Pargyline

RAS: renin-angiotensin-system; CCB: calcium channel blocker
